# Assay of Diastereoisomers of Cefuroxime Axetil in Amorphous and Crystalline Forms Using UHPLC-DAD

**DOI:** 10.1007/s10337-014-2773-y

**Published:** 2014-10-09

**Authors:** Piotr Garbacki, Artur Teżyk, Przemysław Zalewski, Anna Jelińska, Magdalena Paczkowska, Alicja Talczyńska, Irena Oszczapowicz, Judyta Cielecka-Piontek

**Affiliations:** 1 Department of Pharmaceutical Chemistry, Faculty of Pharmacy, Poznan University of Medical Sciences, Grunwaldzka 6, 60-780 Poznan, Poland; 2 Department of Forensic Medicine, Poznan University of Medical Sciences, Święcickiego 6, 60-781 Poznan, Poland; 3 Department of Modified Antibiotics, Institute of Biotechnology and Antibiotics, Starościńska 5, 02-516 Warsaw, Poland

**Keywords:** Column liquid chromatography, Cefuroxime axetil, Diastereoisomers, Amorphous and crystalline forms

## Abstract

A sensitive UHPLC-DAD method was developed for determination of diastereoisomers of cefuroxime axetil in bulk substance in amorphous and crystalline forms as well as in pharmaceutical preparations. Chromatographic separation was achieved on Kinetex C-18 (100 mm × 2.1 mm, 1.7 µm) column with mobile phase consisting of 0.1 % formic acid:methanol (88:12, v/v), at the flow rate of 0.7 mL min^−1^ and total run time of 3 min. The wavelength of the DAD detector was set at 278 nm. Inter-day precision (RSD) was less than 3 % and accuracy level ranged between 98.31 and 104.99 %. Degradation products of cefuroxime axetil in aqueous solutions and in the solid state were identified with a EIS-Q-MS mass spectrometer. The solubility of above-mentioned polymorphic forms of cefuroxime axetil in suitable solvents is a crucial factor during preparation of samples and is essential for chromatographic separation of its diastereoisomers.

## Introduction

**Fig. 1:**
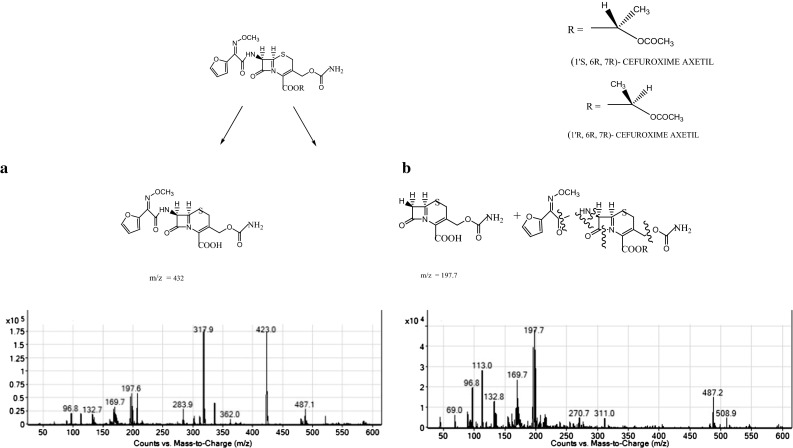
Degradation pathways of cefuroxime axetil in aqueous solutions (**a**) and in solid state (**b**)

Cefuroxime axetil (CA) (Fig. 1) is an orally administered, semi-synthetic acetoxyethyl ester of cefuroxime and it belongs to the second generation of cephalosporins. CA is rapidly hydrolyzed by nonspecific esterase in the intestinal mucosa and blood to the active form, cefuroxime. The drug represents high antibacterial activity against wide range of Gram-positive and Gram-negative microorganisms. CA is mainly used in the treatment of respiratory tract infections [1–4].

Cefuroxime axetil contains three chiral centers and occurs in crystalline and amorphous forms. The stereochemical properties of CA are essential for its bactericidal activity and affinity to biological membranes, while appearance of its polymorphs is important during technological process of obtaining pharmaceutical dosage forms [5, 6].

Cefuroxime axetil is a mixture of two diastereoisomers (A and B). These diastereoisomers can be impurities as well as degradation products of parent drug. The Abbreviated New Drug Application (ANDA) has approved CA as an amorphous dispersion or an amorphous/crystalline mixture following the expiry of patent protection and the appearance of generic products. It is suggested that the crystalline content in the mixture of cefuroxime axetil may “seed” crystallization of the amorphous form and so increases the content of the crystalline form that shows poorer solubility and bioavailability [7].

Chromatographic methods are the most commonly used for the determination of CA and its diastereoisomers in aqueous solutions as well as in the solid state. The proposed conditions of chromatographic separation are based on the employment of ion pairs (e.g., phosphoric acid and its salts) and high content of organic modifier in the mobile phase [6, 8–11].

While the structure of related products of CA has been already described, studies focusing on the development of modern, fast and precise methods of its chromatographic separation, with potential of their application in drug quality control, should be carried out. Different solubility of cefuroxime axetil polymorphs should be taken into account in the preparation of the samples for chromatographic studies. Changes of solubility of samples under applied chromatographic conditions should be also excluded.

The aim of our studies was to develop fast and eco-friendly UHPLC-DAD method allowing the determination of diastereoisomers of cefuroxime axetil in bulk substance as well as in pharmaceutical dosage forms. To achieve these objectives, preparation procedures of amorphous and crystalline CA were implemented.

## Experimental

### Materials

Cefuroxime axetil (amorphous and crystalline forms) was obtained from Institute of Biotechnology and Antibiotics in Warsaw, Poland. Zinnat^®^ coated tablets were manufactured by GlaxoSmithKline (Brentford, Middlesex, Great Britain). Zinoxx^®^ coated tablets were produced by Polfarmex S.A. (Kutno, Poland). Xorimax^®^ coated tablets were originally formulated by Sandoz (Kundl, Austria). All above-mentioned pharmaceutical preparations contained 250 mg of cefuroxime axetil. Other chemicals and reagents were obtained from Merck KGaA (Darmstadt, Germany) and were of chromatographic grade. High-quality pure water was prepared using the Millipore purification system (Millipore, Molsheim, France, model Exil SA 67120).

### Chromatographic Conditions

Chromatographic studies were performed on a Thermo Scientific UHPLC-UltiMate 3000 system. Diastereoisomers of CA were separated with a Kinetex C-18 (100 mm times 2.1 mm, 1.7 µm) column as a stationary phase and a mixture of 0.1 % formic acid:methanol (88:12, v/v), at the flow rate of 0.7 mL min^−1^ as the mobile phase. The wavelength of the DAD detector was set at 278 nm. Separation was carried out at 40 °C. The injected volume was 5 µL. The components of mobile phase and sample solutions were filtered through 0.2 μm nylon membranes. A triple-quadrupole mass spectrometer model 6410B Triple Quad with an electrospray (ESI) interface (both from Agilent Technologies, USA) coupled with chromatograph Agilent 1200 was used for identification of degradation products of cefuroxime axetil. The ESI source of the MS detector was operated in a negative ionization mode. For nebulization, a nitrogen stream at 40 psi (275.8 kPa) was applied. A drying gas, also nitrogen, was delivered at a flow rate of 8 L min^−1^ at 300 °C. The electrospray needle voltage was 4,000 V. The acquisition was carried out in the scan mode, and the spectra were collected in the range of *m/z* 40–600. The MassHunter workstation software (Agilent Technologies, USA) was used for the instrument control, data acquisition and data analysis. The identification of degradation products of cefuroxime axetil based on application of an EIS-Q-MS mass spectrometer was possible using the mobile phase, which composed of 0.1 % formic acid and methanol (50:50, v/v).

### Preparation of Stock Solutions

Cefuroxime axetil powder (4.0 mg) was accurately weighted and transferred to a 10.0 mL volumetric flask. Amorphous form of CA sample was dissolved in a mixture of acetonitrile:water (50:50, v/v), while crystalline form of CA was dissolved in pure methanol. In both cases, final concentration 0.400 mg mL^−1^ of solution was achieved. Stock solutions were stored in darkness at 4 °C and proved stable during the time of the study.

### Preparation of Sample Solution

#### Assay of Cefuroxime Axetil in Bulk Substance

Solutions of cefuroxime axetil in amorphous and crystalline forms were prepared in the same way as stock solutions. The final concentration was also 0.400 mg mL^−1^.

#### Assay of Cefuroxime Axetil in Tablets

The amount of accurately powdered tablets (Zinnat^®^, Zinoxx^®^ and Xormiax^®^) containing 250 mg of cefuroxime axetil was transferred to 50 mL volumetric flasks, dissolved with about 30 mL of mixture of acetonitrile:water (50:50, v/v), mixed in an ultrasound bath for 15 min, filled to volume with the same solvent and filtered. 2.0 mL of so-obtained solutions was transferred to 25 mL volumetric flasks and filled to volume with mixture of acetonitrile:water (50:50, v/v). The final concentration 0.400 mg mL^−1^ was achieved. In addition, pharmaceutical dosage forms of CA (Zinnat^®^, Zinoxx^®^ and Xormiax^®^) were also prepared in analogous manner using pure methanol as a solvent.

### Validation Methodology

The method was validated by evaluation of selectivity, linearity range, precision, accuracy, limit of detection (LOD) and limit of quantification (LOQ). The validation was carried out according to the International Conference of Harmonization Guidelines [12].

#### Selectivity Studies

To evaluate the applicability of proposed UHPLC-DAD chromatographic method in determination of CA in bulk substance and in pharmaceutical dosage forms (Zinnat^®^, Zinoxx^®^ and Xormiax^®^ tablets), degradation studies in conditions of increased temperature and humidity were performed. 5 mg samples of CA in amorphous and crystalline forms were weighted into 5 mL glass vials and exposed to increase relative humidity (RH = 90 %) at 363 K for 12 h. Applied stress conditions simulated possible changes in CA in the solid state during its storage. Zinnat^®^, Zinoxx^®^ and Xormiax^®^ tablets were stored in the same conditions without commercial packages. The identification of degradation products of CA was carried out in aqueous solutions (0.5 mol L^−1^ NaOH at 298 K for 0.5 min) as well as in the solid state (RH = 76 % at 353 K for 6 days).

#### Linearity

To confirm the linearity, eleven standard solutions of CA for both polymorphic forms, in the concentration range 40.0–480.0 μg mL^−1^ (10–120 % of the targeted level of the assay concentration for cefuroxime axetil), were prepared. Each sample was injected in triplicate to evaluate the reproducibility of detector response at each concentration level. The linearity was also investigated for the assay of CA in Zinnat^®^, Zinoxx^®^ and Xormiax^®^ tablets. The same concentration ranges were applied.

#### Precision

Precision of the chromatographic method was determined for three concentrations of cefuroxime axetil (in bulk substance and in tablets): 320.0, 400.0 and 480.0 μg mL^−1^. Each of above-mentioned solutions was injected six times during the same day to evaluate repeatability (intra-day precision) of proposed analytical procedure.

#### Accuracy

To confirm the accuracy of the UHPLC-DAD method, recovery test was performed at three concentration levels: 320.0, 400.0 and 480.0 μg mL^−1^. Known amounts of the reference substance were added at the beginning of the process. Each sample was injected six times.

#### Limits of Detection (LOD) and Quantification (LOQ)

The LOD and LOQ were calculated from the regression equation of the cefuroxime axetil: LOD = 3.3 (*S*_*y*_/*a*), LOQ = 10 (*S*_*y*_/*a*), where *S*_*y*_ is a standard error and *a* is the slope of the corresponding calibration curve.

## Results and Discussion

The separation of diastereoisomers A and B of cefuroxime axetil in amorphous and crystalline polymorphic forms was possible as the result of application of C-18 1.7 μm column as a stationary phase. As the mobile phase, a mixture of 0.1 % formic acid and methanol (88:12, v/v) with flow rate 0.7 mL min^−1^ was used. To evaluate the robustness of proposed chromatographic procedure, the influence of changes of the following parameters on the peak properties was investigated: the composition of mobile phase (concentration of methanol in the range 86–90 %), flow rate (in the range 0.6–0.8 mL min^−1^), absorption wavelength (278 ± 5 nm), and column temperature (40 ± 5 °C). It was observed that column temperature and flow rate of the mobile phase play a crucial role in the separation of diastereoisomers of CA. The most satisfactory resolution was reached when the column temperature was set at 40 °C, with the flow rate 0.7 mL min^−1^.

Proposed chromatographic method represents better separation parameters than pharmacopoeial assay. Recommended values such as relative retention of diastereoisomer B in comparison to the diastereoisomer A, ratio of diastereoisomers and run time of analysis are 1.20, 0.48–0.55 and 25 min for pharmacopoeial method, respectively [10, 13]. While in our studies, we obtained the following values of these parameters: 0.90, 0.75–0.81 and 3 min, respectively. The above-mentioned values of parameters based on UHPLC procedure were established for determination of CA in bulk substance as well as in pharmaceutical preparations (Zinnat^®^, Zinoxx^®^ and Xormiax^®^ tablets).

In comparison to HPLC procedures which have been previously reported for determination of cefuroxime axetil in the presence of its degradation products, the proposed UHPLC-DAD method represents better parameters of chromatographic separation, which is connected with the specification of UHPLC procedure. Its application also provides achievement of more satisfying validation parameters [6, 8, 11].

**Fig. 2:**
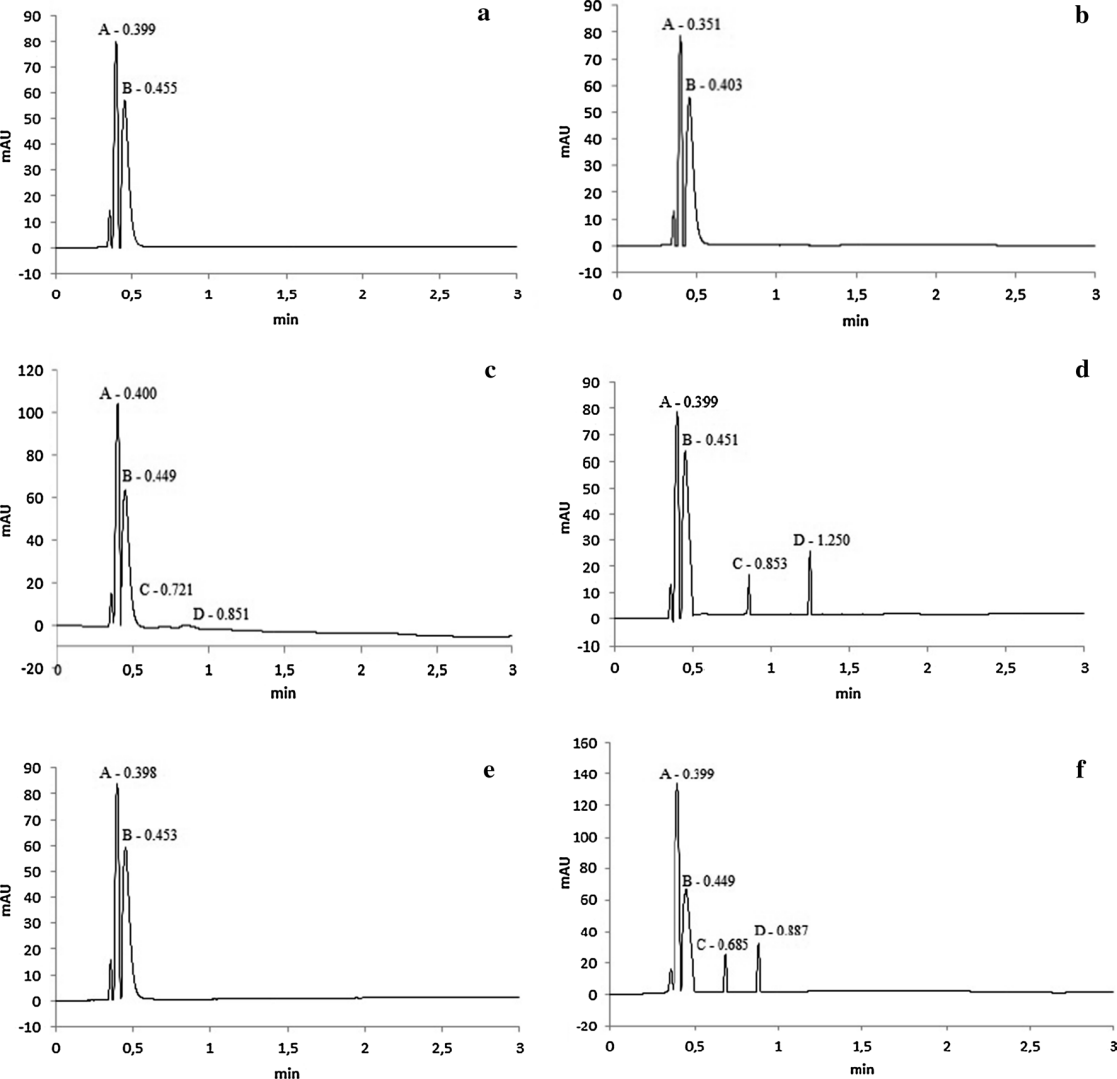
UHPLC chromatograms of non-degraded and degraded samples of CA in bulk substance (amorphous form: **a** and **b**, crystalline form: **c** and **d**) and in tablets (**e** and **f**) (*A* and *B*—diastereoisomers of CA; *C* and *D*—degradation products)

The selectivity of the method for determination of diastereoisomers A and B of cefuroxime axetil in amorphous and crystalline forms was confirmed after dissolution of the samples in the mixture of acetonitrile:water (50:50, v/v) or in pure methanol. The solubility of amorphous and crystalline forms of CA was found to be the highest in above-mentioned suitable solvents. For amorphous form of CA, the most satisfactory parameters of the peaks were observed when the sample was dissolved in the mixture of acetonitrile:water (50:50, v/v) [10]. However, in the case of crystalline form, the best resolution and asymmetry were achieved using pure methanol. Under applied chromatographic conditions, diastereoisomers of cefuroxime axetil in bulk substance were well separated and retention times were: from about 0.35–0.40 min for diastereoisomer A and from 0.40 to 0.45 min for diastereoisomer B (Fig. 2a, b). On chromatograms of degraded samples of cefuroxime axetil, peaks originating from degradation products were observed at retention times from about 0.72 to 1.25 min (Fig. 2c, d). The degradants were not interfering with the main peaks.

To exclude the impact of excipients present in Zinnat^®^, Zinoxx^®^ and Xormiax^®^ and to evaluate the specificity of developed chromatographic method in determination of diastereoisomers A and B, CA selectivity studies for non-degraded and degraded tablets were carried out. The same procedure as in the case of bulk substance was applied. It was observed that after dissolution of tablets containing CA in pure methanol, unsatisfied results were obtained. This may be because the content of the crystalline form of cefuroxime axetil in investigated pharmaceutical preparations is insignificant, and therefore the mixture of acetonitrile:water (50:50, v/v) is a better solvent.

Peaks derived from diastereoisomers of CA in non-degraded tablets represented satisfactory separation and symmetry. Diastereoisomer A was eluted at about 0.4 min, while retention time of diastereoisomer B was about 0.45 min. The parameters of peaks were equal in all studied pharmaceutical formulations (Fig. 2e). In chromatograms obtained from forced degraded tablets, samples peaks originated from degradation products appeared at about 0.68 min and 0.88 min (Fig. 2f). Similarly, in the case of CA in bulk substance, degradants were not interfering with the main peaks.

As expected, the studies of degradation pathways in aqueous solution showed that CA under applied conditions (0.5 mol L^−1^ NaOH at 298 K for 0.5 min) was hydrolysed into its acidic form—cefuroxime. Both diastereoisomers were degraded with similar rate (Fig. 1a). However, in the solid state (RH = 76 % at 353 K for 6 days), the decomposition of CA proceeded multidirectionally, including changes in azabicyclic structure and substituents which can be connected with various degradation rates of diastereoisomers A and B (Fig. 1b).

Spectroscopic purity of peaks of CA in non-degraded and degraded samples (in bulk substance and in tablets) remained unchanged during the stability studies (>98 % purity of the peak) and was sufficient to confirm the selectivity of proposed analytical method. The calibration plots for diastereoisomers A and B of CA, in bulk substance and in Zinnat^®^, Zinoxx^®^, Xormiax^®^ tablets, concentration range in which the method represents the linearity and standard deviation values are summarized in Table 1.

**Table 1:** Regression equations, standard deviation values, LOD and LOQ values of diastereoisomers of cefuroxime axetil in bulk substance and in tablets (concentration range: 0.2–2.4 μg; *n* = 10)

Bulk substance				Tablets					
Amorphous form		Crystalline form		Zinnat^®^		Zinoxx^®^		Xorimax^®^	
A	B	A	B	A	B	A	B	A	B
Regression equation									
*y* = (1103.62 ± 84.14) *x*	*y* = (1561.72 ± 87.06) *x*	*y* = (5026.90 ± 476.85) *x* − 2.5753 ± 0.7652	*y* = (2460.24 ± 79.86) *x* − 0.4008 ± 0.1144	*y* = (2653.69 ± 91.25) *x* − 0.3272 ± 0.1090	*y* = (2360.39 ± 251.99) *x* − 0.5752 ± 0.4266	*y* = (2644.69 ± 134.29) *x* *−* 0.3322 ± 0.1666	*y* = (2312.36 ± 98.60) *x* − 0.4361 ± 0.1261	*y* = (1891.21 ± 121.92) *x* − 0.1486 ± 0.1231	*y* = (2118.38 ± 120.30) *x* − 0.3158 ± 0.1917
Standard deviation values									
0.9956	0.9973	0.9955	0.9992	0.9993	0.9970	0.9981	0.9991	0.9979	0.9984
LOD values (μg)									
0.18	0.19	0.10	0.18	0.08	0.18	0.13	0.10	0.11	0.12
LOQ values (μg)									
0.57	0.59	0.30	0.55	0.2	0.52	0.40	0.30	0.34	0.35

A diastereoisomer A of cefuroxime axetil, B diastereoisomer B of cefuroxime axetil

Precision of the proposed method was characterized by relative standard deviation (RSD, %) which was calculated for six consecutive measurements at three concentration levels: 80 % (*c* = 320 μg mL^−1^), 100 % (*c* = 400 μg mL^−1^) and 120 % (*c* = 480 μg mL^−1^) of standard solutions during the system suitability test. Under developed chromatographic conditions, the investigation of precision was conducted in regards to content of diastereoisomers A and B of cefuroxime axetil in bulk substance in amorphous and crystalline forms and in pharmaceutical preparations (Zinnat^®^, Zinoxx^®^ and Xormiax^®^). The values characterizing inter-day precision are collected in Table 2. All values which characterize the inter-day precision of determination of diastereoisomers of cefuroxime axetil were less than 2 % and meet the criteria required for the precision of the method.

**Table 2:** Inter-day precision and recovery of diastereoisomers of cefuroxime axetil in bulk substance and in tablets

Spiked concentration (μg mL^−1^)	Bulk substance				Tablets					
	Amorphous form		Crystalline form		Zinnat^®^		Zinoxx^®^		Xorimax^®^	
	A	B	A	B	A	B	A	B	A	B
Inter-day precision (RSD, %)										
320	2.13	1.12	2.53	2.83	0.67	0.78	0.60	0.78	0.83	0.51
400	1.54	1.82	1.07	0.63	0.39	1.66	0.68	1.66	0.54	0.88
480	1.64	1.15	2.83	1.88	0.74	0.85	0.77	0.85	0.31	1.30
Recovery (RSD, %)										
320	98.95	98.48	99.99	104.99	99.62	99.66	99.57	100.19	99.64	99.86
400	98.31	99.92	99.40	99.65	100.20	100.40	100.36	100.49	99.78	99.58
480	99.52	99.89	100.02	99.99	100.60	100.41	99.66	99.24	100.02	99.58

A diastereoisomer A of cefuroxime axetil, B diastereoisomer B of cefuroxime axetil

The accuracy of the developed UHPLC-DAD method was evaluated by analyzing contents of diastereoisomers A and B in amorphous and crystalline forms of CA in bulk substance and in pharmaceutical preparations. The average values of the recoveries and standard deviations showed that applied extraction procedure is satisfied and reproducible (Table 2).

LOD and LOQ values were calculated for determination of diastereoisomers A and B of cefuroxime axetil in bulk substance (in both polymorphic forms) and in tablets are shown in Table 1.

## Conclusions

The developed UHPLC-DAD method allows the determination of diastereoisomers A and B of cefuroxime axetil in the presence of its degradation products, in bulk substance (in amorphous and crystalline forms) and in pharmaceutical preparations. The main degradation pathways of CA in aqueous solutions led towards its acidic form and the degradation rates of diastereoisomers A and B were similar. The degradation of CA in the solid state was multidirectional which can be connected with differences in decomposition rates of its diastereoisomers. Thanks to the employment of UHPLC procedure the content of organic modifier was significantly limited (from 38 to 12 %), short run time of analysis (about eight times shorter than in the case of pharmacopoeial method) and satisfactory validation parameters were achieved, what meets the requirements of modern and eco-friendly analytical approaches. Attention, however, focuses on the necessity of using suitable procedure for the preparation of samples containing different polymorphic forms of cefuroxime axetil.
